# Transcatheter aortic valve replacement use in young patients before and after the first low-risk trial publication

**DOI:** 10.1016/j.xjse.2024.100033

**Published:** 2024-11-05

**Authors:** Christina Waldron, Luigi Pirelli, Isaac George, Hiroo Takayama, Arnar Geirsson, Roland Assi, Makoto Mori

**Affiliations:** a Division of Cardiac Surgery, Yale School of Medicine, New Haven, Conn; b Division of Cardiac, Thoracic, and Vascular Surgery, Columbia University, New York, NY

**Figure:**
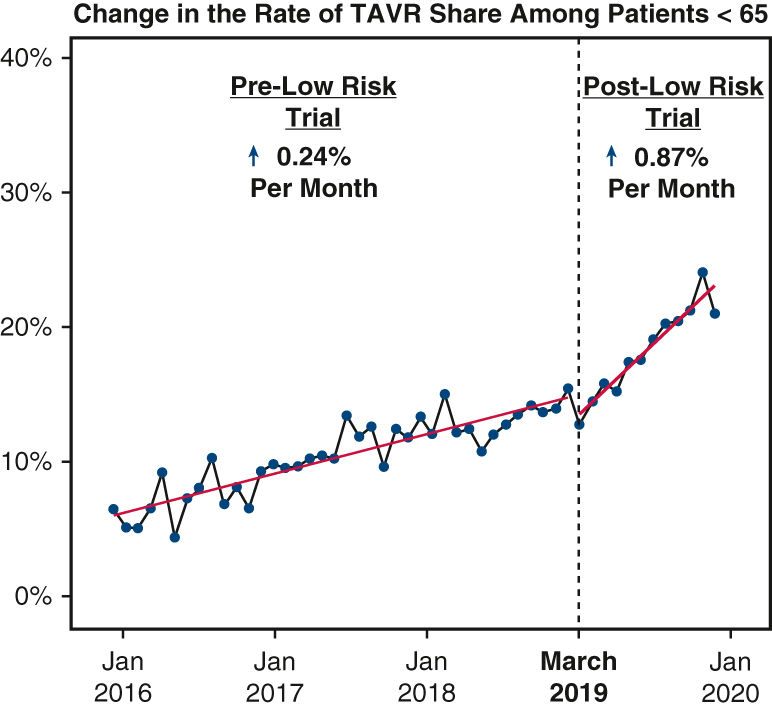
TAVR share accelerated after March 2019, low-risk trials publication.

**Central Message**

TAVR use among young patients accelerated before FDA approval of TAVR in low-risk patients.

Transcatheter aortic valve replacement (TAVR) use in young patients has recently increased.^1^ The high-risk nature of TAVR explant and unknown long-term valve durability are important considerations of TAVR use among young patients with expected longevity.^1^ Although current US guidelines recommend surgical aortic valve replacement (SAVR) in low-risk patients younger than age 65 years,^2^ the first guideline specifying such age threshold was published after US Food and Drug Administration (FDA) approval of TAVR for low-risk patients during 2019. Given that most TAVR trials were conducted among older adults, with low-risk pivotal trials reporting mean age of 73.5 years,^3^^,^^4^ how TAVR use among young patients may have accelerated around this time period is of interest.

To understand how TAVR utilization changed in this age group, we characterized national trends and examined whether or not there were inflection points in TAVR share trend among all AVR procedures performed in young patients.

## Methods

Using National Inpatient Sample data, we conducted a cross-sectional study of patients younger than age 65 years who underwent TAVR, SAVR, or Ross operations between January 1, 2016, and February 29, 2020 (latest available date before COVID-19). Operations and aortic valve pathology were defined using International Classification of Disease 10th edition codes.^5^ Concomitant operations were included; endocarditis was excluded. Change point analysis was used to identify the inflection point in TAVR share during the study period. Difference-in-difference analysis estimated the change in monthly percent TAVR share before and after this inflection point.^5^ We compared in-hospital mortality before and after the inflection point to infer whether or not expansion occurred among lower-risk strata within the TAVR group relative to SAVR.

Difference-in-difference model was specified with outcome being monthly percent of TAVR share among AVRs, covariates of the month as nominal variable, and pre/post indicator of before and after the inflection point. An interaction term was added as the product of month and indicator variable.

Analyses were performed in R version 4.2.2 (R Foundation for Statistical Computing). Yale Institutional Review Board approved this study (#2000028791; December 8, 2023).

## Results

We identified 106,340 AVRs, including 13,095 TAVR (12.3%), 63,620 bioprosthetic SAVR (59.8%), 28,370 mechanical SAVR (26.7%), and 1255 Ross (1.2%). The mean age was 54 ± 10.9 years, including 32,775 (30.8%) women. The proportion of TAVR increased from 6.9% in 2016 to 22.4% in 2020. Mechanical SAVR remained stable from 29.8% to 26%, whereas bioprosthetic SAVR decreased from 62.2% to 50.9%.

**Figure 1:**
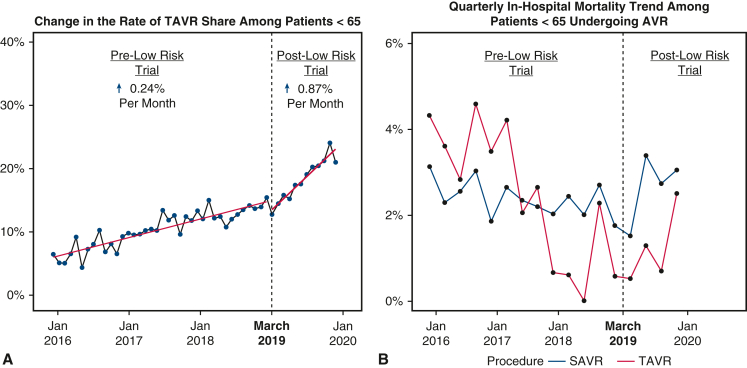
Annual trends of transcatheter aortic valve replacement (*TAVR*) shares and AVR mortality among patients aged younger than 65 years. A, TAVR share accelerated after March 2019. B, Quarterly in-hospital mortality declined in TAVR, whereas mortality remained stable in surgical aortic valve replacement.

Change point analysis identified the inflection point of TAVR share acceleration as March 2019. Before and after March 2019, TAVR share increased at 0.24% ± 0.02% and 0.87% ± 0.12% per month, respectively (interaction term *P* value < .001) (Figure 1, *A*). In TAVR, in-hospital mortality was 2.6% before and 1.0% after March 2019 (*P* = .03) (Figure 1, *B*). In SAVR, in-hospital mortality was 2.6% before and 2.4% after (*P* = .78).

## Discussion

We demonstrated that TAVR use in patients younger than age 65 years increased before FDA approval for low-risk indication, with an inflection point of March 2019, coinciding with low-risk trial publications and preceding FDA approval by 5 months. TAVR share among young patients accelerated after March 2019, tripling the rate of monthly increase. TAVR share among AVRs tripled between 2016 and 2020. Significantly lower in-hospital mortality after March 2019 was observed only among TAVR patients, with SAVR mortality remaining unchanged, highlighting the consistency of their risk-profile throughout the study and suggesting that TAVR use expanded toward lower-risk patients. Several factors may have contributed, including expansion of TAVR toward lower-risk patients and increased utilization of cusp overlap techniques and newer devices. These data suggest the importance of regulatory bodies delineating the indicated age group for novel indication transcatheter valve devices because the first guidance specifying the age threshold lagged by 15 months following FDA approval.

Considerations surrounding patient age are relevant because low risk is not synonymous with young age in prosthetics with age-dependent durability. TAVR share acceleration among young patients around the time of FDA approval may reflect public reception of low-risk approval. Ongoing transcatheter device trials on broader aortic stenosis indications and mitral repair may follow similar trends. As novel devices expand in indications, the momentum of indication creep must be carefully monitored.

The National Inpatient Sample is claims-based and lacks granular risk characteristics. Therefore, not all young patients may be low-risk, and we used in-hospital mortality as a surrogate of patient risk profile.

## Conclusions

Expansion of TAVR use among young, low-risk patients around the publication of low-risk trials suggests that rapid indication expansion preceded FDA approval. This observation may warrant caution in future device regulation processes.

## Conflict of Interest Statement

Dr Geirsson receives consulting fees from Medtronic and Edwards. All other authors reported no conflicts of interest.

The *Journal* policy requires editors and reviewers to disclose conflicts of interest and to decline handling or reviewing manuscripts for which they may have a conflict of interest. The editors and reviewers of this article have no conflicts of interest.
